# Cross-Sectional Associations of Serum Perfluoroalkyl Acids and Thyroid Hormones in U.S. Adults: Variation According to TPOAb and Iodine Status (NHANES 2007–2008)

**DOI:** 10.1289/ehp.1409589

**Published:** 2015-10-30

**Authors:** Glenys M. Webster, Stephen A. Rauch, Nathalie Ste Marie, Andre Mattman, Bruce P. Lanphear, Scott A. Venners

**Affiliations:** 1 Faculty of Health Sciences, Simon Fraser University, Burnaby, British Columbia, Canada; 2 Child and Family Research Institute, BC Children’s Hospital, Vancouver, British Columbia, Canada; 3 Center for Environmental Research & Children’s Health, University of California, Berkeley, Berkeley, California, USA; 4 St. Paul’s Hospital, Vancouver, British Columbia, Canada

## Abstract

**Background:**

Perfluoroalkyl acids (PFASs) are suspected thyroid toxicants, but results from epidemiological studies are inconsistent.

**Objectives:**

We examined associations between serum PFASs and thyroid hormones (THs) in a representative, cross-sectional sample of U.S. adults. We hypothesized that people with high thyroid peroxidase antibodies and low iodine would be more susceptible to PFAS-induced thyroid disruption.

**Methods:**

Our sample included 1,525 adults (≥ 18 years) from the 2007–2008 NHANES study with available serum PFASs and THs. We examined associations between four serum PFASs [perfluorohexane sulfonate (PFHxS), perfluorononanoate (PFNA), perfluorooctanoate (PFOA), and perfluorooctane sulfonate (PFOS)], and serum THs [free triiodothyronine (fT3), free thyroxine (fT4), fT3/fT4, thyroid-stimulating hormone (TSH), total T3 (TT3), and total T4 (TT4)] using multivariable linear regression. We stratified subjects into four groups by two indicators of thyroid “stress”: thyroid peroxidase antibody (TPOAb ≥ 9 IU/mL) and iodine status (< 100 μg/L urine).

**Results:**

Of 1,525 participants, 400 (26%) had low iodine only (T0I1), 87 (6%) had high TPOAb only (T1I0), and 26 (2%) had both high TPOAb and low iodine (T1I1). In general, associations were similar among participants in the groups with neither (T0I0) or only one thyroid stressor (T0I1 or T1I0), suggesting that PFAS–TH associations were not modified by high TPOAb or low iodine alone. However, PFHxS and PFOS were negatively associated (p < 0.05) with fT4, and all four PFASs were positively associated (p < 0.05) with fT3, fT3/fT4, TSH, and TT3 in the group with joint exposure to high TPOAb and low iodine (T1I1).

**Conclusions:**

We found evidence of PFAS-associated thyroid disruption in a subset of U.S. adults with high TPOAb (a marker of autoimmune hypothyroidism) and low iodine status, who may represent a vulnerable subgroup. However, the small sample size, cross-sectional design, and possibility of reverse causation are limitations of this work.

**Citation:**

Webster GM, Rauch SA, Ste Marie N, Mattman A, Lanphear BP, Venners SA. 2016. Cross-sectional associations of serum perfluoroalkyl acids and thyroid hormones in U.S. adults: variation according to TPOAb and iodine status (NHANES 2007–2008). Environ Health Perspect 124:935–942; http://dx.doi.org/10.1289/ehp.1409589

## Introduction

Perfluoroalkyl acids (PFASs), which are used as stain, water, and grease repellents in paper food packaging, carpets, carpet- and upholstery-cleaning liquids, fire-fighting foams, paints, and many other consumer products (Kissa 2001), have been identified as potential thyroid toxicants (Jain 2013; Kim et al. 2011; Lin et al. 2013; Lopez-Espinosa et al. 2012; Melzer et al. 2010; Steenland et al. 2011; Wang et al. 2013, 2014; Webster et al. 2014; Wen et al. 2013; Winquist and Steenland 2014). Nearly 100% of the general population is exposed to PFASs, with detectable levels of perfluorohexane sulfonate (PFHxS), perfluorononanoic acid (PFNA), perflurorooctanoic acid (PFOA), and perfluorooctane sulfonate (PFOS) commonly found in human sera (Health Canada 2010; Kato et al. 2011). In rats, PFOS exposure has been shown to cause hypothyroxinemia, characterized by low thyroxine (T4) without the expected compensatory increase in thyroid-stimulating hormone (TSH) in both pregnant dams and pups (Chang et al. 2008; Lau et al. 2003; Luebker et al. 2005; Thibodeaux et al. 2003; Yu et al. 2009a, 2009b) or hypothyroid effects (low T4 and high TSH) in pups from treated mothers (Luebker et al. 2005). Studies in male and nonpregnant female monkeys also suggest a hypothyroid effect of both ammonium perfluooctanoate (APFO; the ammonium salt of PFOA) and PFOS (Butenhoff et al. 2002; Seacat et al. 2002). In humans, the associations between PFASs and thyroid hormones (THs) are less clear, with some evidence of differences by sex. Melzer et al. (2010) reported that currently treated thyroid disease was associated with PFOA in both men and women (*p* < 0.1 in men) and with PFOS in men only, based on an analysis of 1999–2006 NHANES (National Health and Nutrition Examination Survey) data for the general U.S. population. Another analysis of NHANES data (2007–2010) found that PFASs were associated with increased odds of subclinical hypothyroidism in men (PFOS) and women (PFOA, PFOS, PFHxS), and with both decreased odds of subclinical hyperthyroidism in men (PFOA) and increased odds of subclinical hyperthyroidism in women (PFHxS) (Wen et al. 2013). Similarly, PFOA was associated with both hypothyroidism (both sexes) and hyperthyroidism (women only) in a highly exposed community living downstream of a chemical manufacturing plant (Steenland et al. 2011; Winquist and Steenland 2014), and with parent-reported hypothyroidism in children from the same area (Lopez-Espinosa et al. 2012). There is also growing evidence that serum PFASs are associated with maternal THs during pregnancy and fetal THs in cord blood in a pattern consistent with hypothyroid effects (Kim et al. 2011; Wang et al. 2013, 2014; Webster et al. 2014). However, inconsistent results have been found among studies examining the associations between PFASs and individual THs in nonpregnant adults, with different directions of association, and nonsignificant versus significant associations found for the same thyroid hormones across different populations (Bloom et al. 2010; Dallaire et al. 2009; Emmett et al. 2006; Gilliland 1992; Jain 2013; Ji et al. 2012; Knox et al. 2011; Lin et al. 2013; Olsen et al. 2003; Olsen and Zobel 2007; Wen et al. 2013).

We recently proposed a “multiple hit hypothesis,” postulating that people with multiple thyroid stressors may be particularly susceptible to PFAS-induced thyroid disruption (Webster et al. 2014). In a group of pregnant women who had no prior thyroid-related diagnoses, we found significant positive associations (*p* < 0.05) between serum PFASs and TSH, but only in the small number of women (*n* = 14) with high thyroid peroxidase antibody status [TPOAb ≥ 9 IU/mL serum, a clinical marker of autoimmune hypothyroidism indicating reduced capacity to produce T4 (NHANES 2007b)]. Pregnancy itself also places stress on the thyroid system (Glinoer 1999). The goal of the present study was to test effect modification of PFAS–TH associations by another combination of two thyroid system stressors—high TPOAb status and low urinary iodine status—in a larger, representative sample of U.S. adults. Iodine is an essential nutrient required for the production of T4 in the thyroid gland; about 30% of U.S. adults (≥ 20 years) and 38% of U.S. women of childbearing age (15 to < 45 years) are at least mildly iodine deficient according to World Health Organization guidelines (< 100 μg/L urine) (Caldwell et al. 2011; WHO 2004). We hypothesized that PFASs would be associated with a hypothyroid effect, particularly among people who had high concentrations of serum TPOAb and low urinary iodine levels.

## Methods

### Study Population

We obtained data from the 2007–2008 wave of the NHANES study, a multi-stage, stratified survey using a nationally representative sample to assess the health and nutritional status of the non-institutionalized civilian population of the United States. Subjects provided informed consent and participated in a household interview and a medical exam, during which they provided blood and urine samples for a variety of laboratory analyses. The NHANES study protocol was approved by the National Center for Health Statistics (NCHS) review board, and is described in detail elsewhere (NHANES 2007a).

The overall 2007–2008 NHANES study examined 10,149 children and adults from age 0 to 80 years. THs were analyzed in the 6,917 subjects ≥ 12 years, and PFASs were analyzed in 2,294 of these subjects (NHANES 2007a). From this group, we restricted our study sample to adults ≥ 18 years (*n* = 1,988) with available data for both serum PFASs and TH levels (*n* = 1,798). We excluded 161 participants who had a previous history of diagnosed thyroid disease and a further 10 participants who were currently taking prescription medications known to affect THs. Thyroid active drugs were those with Multum Lexicon category 1, level 1 classification of “thyroid hormones” or “antithyroid agents” and no secondary classification (Multum Lexicon 2015). After further excluding subjects with missing values for the variables race, sex, time of day of serum collection (not used in final models), serum cotinine, current pregnancy, menopause, parity, and urinary iodine, 1,525 participants were included in the final analyses.

### Measurement of Serum PFASs, Thyroid Hormones and Cotinine, and Urinary Iodine

Serum PFAS levels were analyzed at the U.S. Centers for Disease Control and Prevention using online SPE-HPLC-TIS-MS/MS (solid-phase extraction–high performance liquid chromatography–turbo ion spray–tandem mass spectrometry) according to Kuklenyik et al. (2005). Briefly, after dilution with formic acid, a 100 μL aliquot of serum was injected into a commercial column switching system allowing for concentration and chromatographic separation of the analytes. Detection and quantification were performed using tandem mass spectrometry. The constant lower limits of detection (LODs) were 0.1 ng/mL for PFHxS and PFOA, 0.082 ng/mL for PFNA, and 0.2 ng/mL for PFOS (NHANES 2007b). Of the 12 polyfluorinated compounds analyzed in sera, we restricted our analysis to 4 chemicals—PFHxS, PFNA, PFOA, and PFOS—which were detected in > 99% of serum samples. Values below the limit of detection were replaced with LOD divided by the square root of 2.

Serum THs were measured at the University of Washington (Seattle, WA, USA) using the Access Free T3 (triiodothyronine) (fT3), total T3 (TT3), and total T4 (thyroxine) (TT4) competitive binding immunoenzymatic assays, the Access Free T4 (fT4) two-step enzyme immunoassay, and the HYPERsensitive human thyroid-stimulating hormone (hTSH) assay. Serum TPOAb was measured by the Access two-step immunoenzymatic “sandwich” assay. Urinary iodine and serum cotinine concentrations were determined by ICP-DRC-MS (inductively coupled plasma mass spectrometry) and ID HPLC-APCI MS/MS (isotope-dilution high-performance liquid chromatography/atmospheric pressure chemical ionization tandem mass spectrometry) respectively (NHANES 2007b).

### Statistical Modeling

We developed separate multivariable linear models to test associations between interquartile ratio increases (IQ ratio = 75th/25th percentiles of PFAS levels) for serum PFHxS, PFNA, PFOA, and PFOS and levels of the THs fT3, fT4, the fT3/fT4 ratio, TSH, TT3, and TT4. We examined fT3/fT4 to investigate whether PFAS–TH associations were consistent with increased T4 to T3 conversion, and increased hepatic T4 clearance—two mechanisms by which PFASs have been proposed to alter TH levels (Chang et al. 2008; Yu et al. 2009a). All PFASs and THs were natural log transformed because these models best met the assumptions of normally distributed and homoscedastic residuals. To evaluate variation in associations according to thyroid stressors, we examined models in four strata of the population using clinical cutoffs for autoimmune hypothyroidism (TPOAb normal: < 9 vs. high: ≥ 9 IU/mL serum) (NHANES 2007b) and low urinary iodine status (normal: ≥ 100 vs. low: < 100 μg/L urine) (WHO 2004). We investigated the role of influential subjects (identified as dfβ > 2.0) on model stability. In sensitivity analyses, we examined whether adding education or income, or removing pregnant women from the models changed our results. We also explored effect modification by sex within each stratum in a secondary analysis. We assumed linearity but were unable to confirm it because of the low number of participants in the high TPOAb and low iodine group.

We present the magnitudes of association as the average percentage difference in TH level for an IQ ratio contrast of serum PFAS, calculated as [(IQ Ratio^Beta^) – 1] × 100. This approach scales the magnitudes of association to an exposure range that is present in the study population.

### Covariate Selection and Stratification Variables

We used a directed acyclic graph (DAG) (see Figure S1) to identify potential confounding variables in the causal association between PFASs and THs. Variables were included in the model if they were considered to be causally associated with both exposure and outcome. Because smoking is thought to affect thyroid function (Asvold et al. 2007), we also included serum cotinine because it may help to improve model precision. Final models included a single PFAS and were adjusted for continuous measures of age and log_10_-transformed serum cotinine, as well as race/ethnicity, sex, parity, pregnancy, and menopause status (Table 1). Body mass index (BMI) was not included in the models because we interpreted it to be causally “downstream” of THs (Pearce 2012) and PFOA (Halldorsson et al. 2012; Johnson et al. 2014), both of which may affect body weight; adjusting for BMI would therefore bias the models (Cole et al. 2010). Although data were available for urinary perchlorate (another suspected thyroid toxicant), we did not adjust for perchlorate because there was no reason to suspect that it would be systematically associated with PFASs (e.g., a common exposure source). Also, log-transformed perchlorate and PFAS levels were poorly correlated in our data set (Pearson *r* < 0.05). Models were further stratified by both TPOAb status (normal: < 9 IU/mL or high: ≥ 9 IU/mL serum) and iodine status (normal: ≥ 100 μg/L or low: < 100 μg/L urine), yielding results for four population subgroups: normal TPOAb and iodine (T0I0); low iodine only (T0I1); high TPOAb only (T1I0); and high TPOAb, low Iodine (T1I1).

**Table 1:** Serum PFAS levels [geometric means (GM) or means] and serum thyroid hormone levels by population characteristics in our study sample (U.S. adults from NHANES 2007–2008) (*n *= 1,525).

Variable	Study sample	T1I1^*a*^^,^^*b*^	PFHxS (ng/mL)	PFNA (ng/mL)	PFOA (ng/mL)	PFOS (ng/mL)	Free T3 (pg/mL)	TT3 (ng/dL)	Free T4 (ng/dL)	TT4 (μg/dL)	TSH (mIU/L)
	*n *(%^*c*^)	*n *(%^*d*^)	GM^*e*^	GM^*e*^	GM^*e*^	GM^*e*^	Mean	Mean	Mean	Mean	GM
All	1,525 (100)	26 (100)	1.9	1.5	4.2	13.5	3.2	13.3	0.8	7.8	1.5
Sex
Male	813 (53)	7 (27)	2.6	1.4	4.7	17.1	3.3	114.4	0.8	7.5	1.5
Female	712 (47)	19 (73)	1.4	1.1	3.5	10.9	3.1	112.5	0.8	8.0	1.6
Age (years)^*f*^
18–39	579 (38)	6 (23)	1.6	1.2	3.8	10.9	3.4	119.3	0.8	7.8	1.4
40-64	617 (40)	14 (54)	1.9	1.3	4.2	14.7	3.2	113.9	0.8	7.6	1.6
≥ 65	329 (22)	6 (23)	2.6	1.3	4.4	19.0	3.0	102.3	0.8	8.0	1.8
Race/ethnicity
White	688 (45)	17 (65)	2.1	1.2	4.4	14.4	3.2	111.4	0.8	7.6	1.7
Black	293 (19)	3 (12)	2.3	1.5	4.4	18.7	3.2	109.6	0.8	7.6	1.2
Hispanic	463 (30)	5 (19)	1.6	1.1	3.6	10.9	3.3	119.3	0.8	8.1	1.5
Other	81 (5)	1 (4)	1.5	1.4	3.7	13.7	3.2	111.9	0.8	7.9	1.5
Serum cotinine (ng/mL)^*f*^^,^^*g*^
≤ DL	230 (15)	2 (8)	1.8	1.1	3.7	13.6	3.2	112.3	0.8	8.0	1.6
DL to 4.9	835 (55)	20 (77)	1.9	1.3	4.1	14.2	3.2	112.0	0.8	7.8	1.6
≥ 5.0	460 (30)	4 (15)	2.1	1.3	4.3	13.4	3.3	116.8	0.8	7.6	1.4
Parity (previous births)^*h*^
0	184 (26)	5 (26)	1.3	1.0	3.4	9.4	3.2	116.7	0.8	8.0	1.5
1	94 (13)	2 (11)	1.3	1.1	3.3	9.5	3.1	112.9	0.8	8.2	1.4
2	187 (26)	5 (26)	1.4	1.2	3.7	12.4	3.1	110.0	0.8	8.0	1.6
≥ 3	247 (35)	7 (37)	1.4	1.1	3.4	11.6	3.1	111.0	0.8	8.1	1.6
Pregnancy status^*h*^
Not pregnant	705 (99)	18 (95)	1.4	1.1	3.5	11.0	3.1	112.0	0.8	8.0	1.6
Pregnant	7 (1)	1 (5)	0.5	0.6	1.6	4.5	2.9	155.7	0.6	9.7	1.9
Menopause status^*h*^
Premenopause	402 (56)	7 (37)	1.0	1.0	3.0	8.7	3.2	116.9	0.8	8.0	1.5
Postmenopause	310 (44)	12 (63)	2.0	1.3	4.1	14.7	3.0	106.7	0.8	8.2	1.7
TPOAb status (IU/mL)
Normal (< 9)	1,412 (93)	0 (0)	2.0	1.2	4.1	13.9	3.2	113.6	0.8	7.6	1.5
High (≥ 9)	113 (7)	26 (100)	1.7	1.2	3.7	13.3	3.1	111.4	0.8	7.7	2.1
Iodine status (μg/L)
Normal (> 100)	1,099 (72)	0 (0)	2.0	1.3	4.2	14.2	3.2	113.1	0.8	7.7	1.5
Low (≤ 100)	426 (28)	26 (100)	1.8	1.1	3.8	13.0	3.2	114.4	0.8	7.9	1.6
Subgroups by TPOAb & iodine status^*a*^^,^^*b*^
T0I0	1,012 (66)	— (—)	2.0	1.3	4.3	14.3	3.2	113.2	0.8	7.7	1.5
T0I1	400 (26)	— (—)	1.9	1.1	3.8	13.1	3.2	114.8	0.8	7.9	1.6
T1I0	87 (6)	— (—)	1.7	1.2	3.9	14.1	3.2	112.3	0.8	7.7	1.9
T1I1	26 (2)	— (—)	1.9	1.0	3.1	11.0	3.1	108.4	0.8	7.6	2.6
^***a***^T0I0: normal TPOAb and iodine; T0I1: low iodine only; T1I0 high TPOAb only; T1I1: high TPOAb and low iodine. ^***b***^TPOAb cutoffs: normal: < 9, high: ≥ 9 IU/mL serum. Iodine cutoffs: normal ≥ 100, low: < 100 μg/L urine. ^***c***^Percent in study sample. ^***d***^Percent in T1I1. ^***e***^All PFASs were detected in > 99% of study serum samples. Constant lower limits of detection (LODs, ng/mL) = 0.1 (PFHxS, PFNA, and PFOA) and 0.2 (PFOS). Values < LOD were replaced with LOD divided by the square root of 2. Geometric means (GMs) include these imputed values. ^***f***^Treated continuously in models. ^***g***^Detection limit (DL; 0.011 ng/mL, serum cotinine). ^***h***^Calculated in women only.											

All analyses were performed with SAS version 9.3 (SAS Institute Inc.), and used the SURVEYREG, SURVEYFREQ, and SURVEYMEANS procedures to account for NHANES’ complex survey design and weighting. Alpha values of 0.05 and 0.1 were used as the criteria for statistical significance for PFAS terms and PFAS × sex interaction terms, respectively.

## Results

### PFAS Levels by Population Characteristics and Subgroup

We restricted our analysis to PFHxS, PFNA, PFOA, and PFOS, which were detected in > 99% of serum samples. Across the whole study population, geometric means (GMs; nanograms per milliliter) and IQ ratios for serum PFASs were 1.9 and 3.2 (PFHxS); 1.5 and 2.1 (PFNA); 4.2 and 2.1 (PFOA); and 13.9 and 2.5 (PFOS) (Table 2). All PFASs were moderately and significantly correlated with each other (Spearman rho = 0.41 to 0.67, *p* < 0.001) (see Table S1). PFAS levels were higher in men than in women. Within women, PFAS levels were higher in nonpregnant and postmenopausal women (Table 1). PFAS levels did not decrease with parity. In general, PFAS levels were similar or lower in participants with high versus normal TPOAb, and were also lower in participants with low versus normal iodine. When the population was stratified by both TPOAb and iodine status, PFAS levels (except PFHxS) were also lower in T1I1 compared to the other three subgroups. TH levels also varied across many population characteristics (Table 1). Participants in the T1I1 group were more likely to be white, female, middle-aged (40–64 years), postmenopausal, and with intermediate serum cotinine levels (detection limit to 4.9 ng/mL) than was typical in the study sample (Table 1).

**Table 2:** Summary of serum PFAS and thyroid hormone levels, and urinary iodine levels in our study sample (*n *= 1,525 U.S. adults, NHANES 2007–2008).

Variable	Units	Minimum	5th percentile	25th percentile	Median	75th percentile	95th percentile	Maximum	GM	Mean	IQ ratio^*a*^
Serum PFAS
PFHxS	ng/mL	< LOD^*b*^	0.4	1.1	2.0	3.5	9.2	81.6	1.9	3.0	3.2
PFNA	ng/mL	< LOD^*b*^	0.5	0.8	1.2	1.7	3.4	25.7	1.5	1.5	2.1
PFOA	ng/mL	< LOD^*b*^	1.6	2.9	4.2	6.0	9.5	104.0	4.2	4.8	2.1
PFOS	ng/mL	< LOD^*b*^	3.8	8.8	14.2	22.4	45.5	253.0	13.9	18.6	2.5
Thyroid hormone
Free T3	pg/mL	1.9	2.6	3.0	3.2	3.4	3.9	6.4	NA^*c*^	3.2	—
Free T4	ng/dL	0.4	0.6	0.7	0.8	0.9	1.0	2.5	NA^*c*^	0.8	—
FT3/FT4	—	1.9	3.0	3.7	4.1	4.7	5.7	12.6	NA^*c*^	4.2	—
TSH	μIU/mL	0.0	0.5	1.1	1.6	2.4	4.1	61.5	1.5	2.0	—
TT3	ng/dL	58.0	80.0	99.0	112.0	126.0	150.0	311.0	NA^*c*^	113.5	—
TT4	μg/dL	3.2	5.5	6.8	7.6	8.7	10.4	18.9	NA^*c*^	7.8	—
Urinary iodine
TPOAb	IU/mL	0.1	0.1	0.3	0.6	1.3	43.4	965.2	0.8	13.8	—
Iodine	μg/L	11.5	39.3	92.6	157.5	258.8	634.5	50502.0	159.4	309.7	—
^***a***^Interquartile ratio (IQ ratio) = 75th/25th percentile for serum PFASs. ^***b***^Constant lower limits of detection (LODs; ng/mL) = 0.1 (PFHxS, PFNA, and PFOA) and 0.2 (PFOS). All 4 PFASs were detected in > 99% of sera. Values < LOD were replaced with LOD divided by the square root of 2. ^***c***^Normally distributed variables; geometric means not reported (NA). All other variables were log-normally distributed.											

### Thyroid Hormone and Iodine Levels

Of the 1,525 participants, 113 (8%) had TPOAb above the clinical cutoff (≥ 9 IU/mL), and 426 (28%) were at least mildly iodine deficient according to World Health Organization criteria (< 100 μg/L urine) (WHO 2004) (Table 1). When the study sample was stratified by both iodine and TPOAb status, 1,012 participants (66%) had normal TPOAb and iodine (T0I0), 400 (26%) had low iodine only (T0I1), 87 (6%) had high TPOAb only (T1I0), and 26 (2%) had both high TPOAb and low iodine (T1I1). Several participants had TH levels suggestive of undiagnosed primary hypothyroidism (fT4 < 0.6 ng/dL and TSH > 5.6 mIU/L, *n* = 3), subclinical hypothyroidism (0.6 ≤ fT4 ≤ 1.6 ng/dL, and TSH > 5.6 mIU/L, *n* = 21, including 2 participants in the T1I1 group), primary hyperthyroidism (fT4 > 1.6 ng/dL and TSH < 0.34 mIU/L, *n* = 3), or subclinical hyperthyroidism (0.6 ≤ fT4 ≤ 1.6 ng/dL, fT3 ≤ 3.9 pg/mL and TSH < 0.34 mIU/L, *n* = 16) using NHANES 2007–2008 clinical cutoffs (NHANES 2007a). These individuals were retained in all models.

### PFASs versus Thyroid Hormone Associations

**Figure 1:**
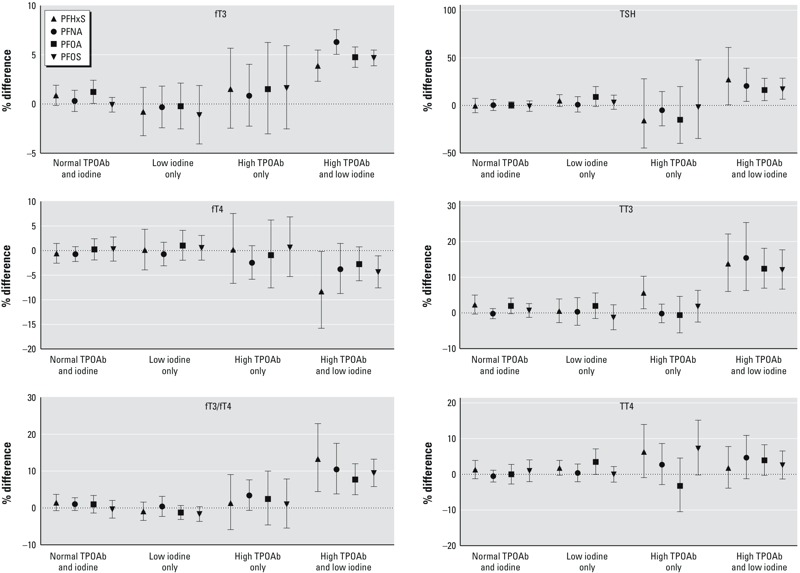
Percent differences in serum thyroid hormone levels for an interquartile ratio increase in Ln serum PFAS concentrations in U.S. adults (NHANES 2007–2008). Results are stratified by TPOAb status (normal < 9; high, ≥ 9 IU/mL serum) and iodine status (normal, ≥ 100; low, < 100 μg/L urine). Error bars represent the 95% CIs. Models are adjusted for age, race, log serum cotinine, sex, parity, pregnancy, and menopause status. Interquartile ratios: 3.2 (PFHxS), 2.1 (PFNA), 2.1 (PFOA), 2.5 (PFOS). PFASs and THs were Ln-transformed in models. Percent differences = [(IQ Ratio^Beta^) – 1] × 100.

We found few statistically significant associations (*p* < 0.05) in the groups with normal TPOAb and iodine (T0I0) or in those with low iodine or high TPOAb alone (T0I1 and T1I0). Weak positive associations were found for PFOA and fT3 (1%) in T0I0, and PFHxS and TT3 (6%) in T1I0 (Table 3). In contrast, all four PFASs were consistently and positively associated (*p* < 0.05) with fT3, fT3/fT4, TSH, and TT3 in the group with both high TPOAb and low urinary iodine (T1I1) (Table 3, Figure 1). PFHxS and PFOS were negatively associated (*p* < 0.05) with fT4 in T1I1, but TT4 associations were not significantly different from the null. For each doubling to tripling (IQ ratio contrast) in serum PFAS levels, the percent difference in TH levels in the sample ranged from 4% to 6% (fT3), 8% to 13% (fT3/fT4), 16% to 27% (TSH), and 12% to 15% (TT3), and –4% to –8% (fT4, PFHxS and PFOS only) in the T1I1 group (Table 3, Figure 1).

**Table 3:** Estimated percent difference (% diff)*^a^* (95% CIs) in serum thyroid hormone levels in U.S. adults for each interquartile ratio (IQ ratio) increase in serum PFAS concentrations*^b^* ^,^ *^c^* stratified by urinary iodine and serum TPOAb status.*^d^*

TH	PFAS	T0I0^*e*^ (*n *= 1,012)	T0I1^*e*^ (*n *= 400)	T1I0^*e*^ (*n *= 87)	T1I1^*e*^ (*n *= 26)
		% diff (95% CI)	% diff (95% CI)	% diff (95% CI)	% diff (95% CI)
fT3	PFHxS	0.9 (–0.1, 1.9)	–0.8 (–3.2, 1.7)	1.5 (–2.5, 5.7)	3.9 (2.3, 5.5)**
	PFNA	0.3 (–0.8, 1.4)	–0.3 (–2.4, 1.8)	0.8 (–2.2, 4)	6.3 (5, 7.5)**
	PFOA	1.2 (0.1, 2.4)**	–0.2 (–2.5, 2.1)	1.5 (–3, 6.3)	4.8 (3.7, 5.8)**
	PFOS	–0.1 (–0.8, 0.7)	–1.1 (–4, 1.9)	1.6 (–2.5, 5.9)	4.7 (3.9, 5.5)**
fT4	PFHxS	–0.6 (–2.6, 1.5)	0.1 (–3.9, 4.3)	0.2 (–6.7, 7.5)	–8.3 (–15.8, –0.2)**
	PFNA	–0.7 (–2.2, 0.8)	–0.7 (–3.1, 1.7)	–2.5 (–5.8, 1)	–3.8 (–8.7, 1.4)
	PFOA	0.2 (–1.9, 2.4)	1.0 (–2, 4.1)	–0.9 (–7.6, 6.2)	–2.7 (–6.1, 0.8)
	PFOS	0.3 (–2.1, 2.8)	0.5 (–1.9, 3.1)	0.6 (–5.3, 6.9)	–4.4 (–7.6, –1.1)**
fT3/fT4	PFHxS	1.5 (–0.7, 3.7)	–0.9 (–3.4, 1.6)	1.3 (–5.9, 9.1)	13.3 (4.4, 22.9)**
	PFNA	1.0 (–0.7, 2.8)	0.4 (–2.3, 3.2)	3.4 (–0.7, 7.6)	10.5 (3.8, 17.5)**
	PFOA	1.0 (–1.4, 3.4)	–1.2 (–3.1, 0.7)	2.4 (–4.6, 10)	7.7 (3.6, 12)**
	PFOS	–0.4 (–2.7, 2.1)	–1.7 (–3.6, 0.4)	1.0 (–5.4, 7.9)	9.5 (5.8, 13.2)**
TSH	PFHxS	–0.3 (–7.6, 7.5)	5.0 (–1, 11.3)	–15.8 (–44.7, 28.1)	27.3 (0.7, 60.9)**
	PFNA	0.3 (–5.3, 6.2)	0.9 (–6.9, 9.3)	–5.0 (–21.3, 14.6)	20.5 (4.3, 39.1)**
	PFOA	0.3 (–3, 3.8)	8.9 (–1, 19.9)	–15.1 (–39.8, 19.9)	16.2 (5.1, 28.5)**
	PFOS	–0.8 (–6.2, 4.9)	3.2 (–4, 10.8)	–1.7 (–34.7, 47.9)	17.1 (6.6, 28.7)**
TT3	PFHxS	2.3 (–0.3, 5)	0.6 (–2.7, 3.9)	5.6 (1.2, 10.3)**	13.8 (6, 22.1)**
	PFNA	–0.2 (–1.6, 1.2)	0.3 (–3.5, 4.3)	–0.2 (–2.7, 2.5)	15.4 (6.3, 25.3)**
	PFOA	2.0 (–0.2, 4.2)	2.0 (–1.5, 5.6)	–0.6 (–5.6, 4.6)	12.4 (7, 18.1)**
	PFOS	0.7 (–1.2, 2.6)	–1.3 (–4.7, 2.3)	1.8 (–2.6, 6.3)	12.0 (6.7, 17.7)**
TT4	PFHxS	1.3 (–1.2, 3.9)	1.8 (–0.3, 3.9)	6.3 (–0.9, 14)	1.8 (–3.9, 7.8)
	PFNA	–0.5 (–2.2, 1.2)	0.4 (–2.1, 2.9)	2.7 (–2.9, 8.6)	4.7 (–1.2, 10.9)
	PFOA	0.0 (–2.7, 2.8)	3.5 (–0.1, 7.1)	–3.2 (–10.5, 4.6)	3.9 (–0.3, 8.3)
	PFOS	1.0 (–2, 4)	0.0 (–2.2, 2.2)	7.2 (–0.2, 15.2)	2.5 (–1.3, 6.5)
^***a***^PFASs and THs were Ln-transformed in models. Percent differences = [(IQ ratio^Beta^) – 1] × 100. ^***b***^Interquartile ratio = 75th/25th percentiles of serum PFASs: 3.2 (PFHxS), 2.1 (PFNA), 2.1 (PFOA), 2.5 (PFOS). ^***c***^Models are adjusted for age, race, log serum cotinine, sex, parity, pregnancy, and menopause status. ^***d***^TPOAb cutoffs: normal: < 9, high: ≥ 9 IU/mL serum. Iodine cutoffs: normal ≥ 100, low: < 100 μg/L urine. ^***e***^T0I0: normal TPOAb and iodine; T0I1: low iodine only; T1I0: high TPOAb only; T1I1: high TPOAb and low iodine. **p < 0.05.					

To compare associations across different numbers of thyroid stressors, we re-plotted the results in Figure S2. Overall, findings did not support modification of PFAS–TH associations by exposure to high TPOAb or low iodine alone. For all PFAS and TH combinations, point estimates for the T0I1 or T1I0 groups were similar to those in the T0I0 group and/or there was considerable overlap in 95% confidence intervals (CIs) among the three groups. However, associations for fT3, fT3/fT4, TSH (PFOA and PFOS only), and TT3 were clearly higher (higher point estimates and non-overlapping CIs) in T1I1 than in T0I0. Although qualitative, these comparisons suggest that PFAS–TH associations are modified by joint exposure to high TPOAb and low iodine, but not by exposure to either thyroid stressor alone.

In influential points analysis in the T1I1 group, one participant had dfβ > 2.0 in 9 of the 24 T1I1 models. When this participant was removed from the models, only 8 of the previous 18 significant associations persisted (*p* < 0.05), including 1 that changed direction (fT3 and PFHxS, *p* < 0.05) (see Table S3). However, the remaining 7 associations (including for fT4, fT3/fT4, TSH, and TT3) were strengthened (20% to 550%), although estimates were also less precise. A new negative association (*p* < 0.05) between PFOS and TT4 was also found. Overall, these results support our general findings of positive associations between certain PFASs and fT3/fT4, TSH, and TT3, and negative associations with fT4 in the T1I1 group, but also underline the need for cautious interpretation of results based on few observations.

In sensitivity analyses, adding income (continuous measure of the household income to poverty ratio) or maternal education (≤ high school vs. > high school) or removing pregnant women from the adjusted models did not affect our conclusions (results not shown). In each case, the statistically significant (*p* < 0.05) associations reported in Table 3 remained significant or became marginally significant (*p* < 0.1 for two models including income), and nearly all model estimates in the T1I1 group changed by < 10%. The only exception was an 18%–31% strengthening of the fT3, fT4, and fT3/fT4 associations in the T1I1 group when income was added to the model (results not shown). Because there is no reason to expect a causal association between income and THs (see Figure S1), we decided to leave income out of our main models.

In sex-stratified models, we found evidence of sex-specific associations in 31 of 96 (32%) models (*p* < 0.1 for the PFAS × sex interaction term), most of which were in the T1I1 group (*n* = 19) (see Table S2 and Figure S3). However, the interpretation of these results is limited by the very small sample sizes in this group (*n* = 7 men and *n* = 19 women). Briefly, associations in T1I1 women mirrored the pattern found in the combined analysis (positive associations with fT3, fT3/fT4, and TSH, and negative with fT4). In T1I1 men, PFAS associations with fT4, fT3/fT4, and TSH were stronger but less precise than in T1I1 women. However, associations were negative for men but positive for women in several cases, that is, for fT3 and TT4 in the T1I1 group, and for TT3 in the T0I1 group (see Table S2 and Figure S3). These results raise questions about differences in PFAS–TH associations by sex, and deserve additional attention in future studies.

## Discussion

We found evidence of PFAS-associated thyroid disruption in the subset of U.S. adults with joint exposure to both high TPOAb and low urinary iodine levels (T1I1) but limited evidence in the rest of the population. Approximately 1.3% of U.S. adults are expected to fall into the T1I1 group after accounting for those excluded from our study sample (i.e., [26/(1,988)] × 100). In the T1I1 subgroup, a doubling to tripling (IQ ratio contrast) in serum PFAS concentrations was associated with modest increases in fT3 (4% to 6%), fT3/fT4 ratios (8% to 13%), TSH (16% to 27%), TT3 (12% to 15%) and modest decreases in serum fT4 (–3% to –8%). Only two statistically significant associations were observed in the other three population subgroups; both were weaker but in the same direction as those found in T1I1. In a qualitative comparison of associations across population subgroups, PFAS associations were modified by joint exposure to high TPOAb and low iodine, but not by exposure to either thyroid stressor alone. Though based on a small sample size, these results suggest that people with both high TPOAb and low urinary iodine may be more vulnerable to PFAS-induced thyroid disruption than the rest of the population.

To the best of our knowledge, this is the first study to examine the modifying effects of both high TPOAb and low urinary iodine on associations between THs and any environmental toxicant. We recently reported positive associations between PFASs and TSH (*p* < 0.05) in pregnant women (pregnancy is another thyroid stressor) with high TPOAb (≥ 9 IU/mL), but not in women with normal TPOAb (< 9 IU/mL) (Webster et al. 2014). Urinary iodine levels were not available for that study. Several other studies have examined effect modification by iodine status alone. Blount et al. (2006) reported stronger associations between perchlorate and THs (inverse associations with TT4 and positive associations with TSH) in U.S. women with low versus normal urinary iodine levels (< 100 μg/L vs. ≥ 100 μg/L urine). Similarly, stronger inverse associations were found with maternal urinary bisphenol A (BPA) and newborn TSH (in girls only) in mothers with low versus normal urinary iodine status at 26 weeks gestation (< 150 μg/g vs. ≥ 150 μg/g creatinine-adjusted) in a Cincinnati, Ohio, birth cohort (Romano et al. 2015). In contrast, daily maternal iodine intake (assessed by questionnaire) did not modify associations between maternal urinary BPA with maternal or newborn THs in the CHAMACOS (Center for the Health Assessment of Mothers and Children of Salinas) birth cohort (*n* = 335 and 364, respectively) (Chevrier et al. 2013). Taken together, these studies support the hypothesis that populations with preexisting thyroid stressors may be more susceptible to thyroid-disrupting chemicals. It would be worthwhile to test this “multiple hit hypothesis” further in a larger sample, and in subpopulations with combinations of thyroid stressors such as undiagnosed or untreated TH disease, low iodine status, and pregnancy. Effect modification by TH stressors may also be relevant for other thyroid-disrupting chemicals, both singly and in complex mixtures, and should be explored in future studies.

Although we found limited evidence of PFAS-associated thyroid disruption in most of the U.S. population (i.e., in the T0I0, T0I1, and T1I0 groups), the directions of association found in the T1I1 group are generally consistent with other recent U.S. studies. Wen et al. (2013) found positive associations between PFHxS, PFOA, or PFOS and subclinical hypothyroidism in men or women using NHANES 2007–2008 data. PFOA was also associated with hypothyroidism in men (Winquist and Steenland 2014) and women (Steenland et al. 2011) in highly exposed communities living downstream of a chemical plant in the mid-Ohio valley; similarly, PFOA was associated with parent-reported thyroid disease or hypothyroidism in children from the same communities (Lopez-Espinosa et al. 2012). However, the same studies reported associations with hyperthyroidism in women (PFOA) (Winquist and Steenland 2014) and subclinical hyperthyroidism in women (PFHxS) (Wen et al. 2013), which is inconsistent with our T1I1 results.

Similar to our findings in the T1I1 group, PFASs have been associated with lower fT4 or higher TSH in pregnant women or their newborns (Chan et al. 2011; Kim et al. 2011; Wang et al. 2013, 2014), but also with lower TT3 and TT4 (Kim et al. 2011; Wang et al. 2014), which is contrary to our T1I1 findings in U.S. adults. Other studies of adolescents and adults report inconsistent associations between PFASs and THs. PFOA was positively related to TSH in one study of occupationally exposed workers (Gilliland 1992) but not in another (Olsen and Zobel 2007). In contrast to our T1I1 findings, PFOS was negatively related to TSH in Inuit adults from Northern Canada (Dallaire et al. 2009). No association was found between serum PFOA and either abnormal TSH levels or a history of thyroid disease in residents of the Little Hocking water district, an area in southeastern Ohio with a known PFOA-contaminated water supply (Emmett et al. 2006). In contrast to our T1I1 findings, PFOS was positively related to fT4 in the Canadian Inuit study (Dallaire et al. 2009). Free T4 levels also increased significantly across categories of PFNA in adolescents and young adults from Taiwan (Lin et al. 2013). Differences in study population, study design, PFAS exposure levels, and relatively high co-exposures to other thyroid disrupting chemicals—such as polychlorinated biphenyls (PCBs)—may help to explain some of these discrepancies across studies. Our study suggests that the prevalence of effect modifiers, such as pregnancy, iodine status, and TPOAb status, may also help to explain these apparent differences across study populations.

### Sex-Specific Findings

Our results also suggest that PFAS–TH associations may differ by sex, although interpretation is limited by small sample sizes in the T1I1 group (*n* = 7 men and *n* = 19 women). We found significant PFAS × sex interactions (*p* < 0.1) in 31 of 96 models (32%), including in 19 of 24 (79%) of the T1I1 models (see Table S2 and Figure S3). In general, associations in T1I1 women mirrored those in the combined T1I1 group, whereas associations in T1I1 men were stronger but less precise (fT4, fT3/fT4, and TSH), or in the opposite direction (fT3 and TT4) compared with women. It is noteworthy that sex-specific associations have been reported elsewhere, including in U.S. adults (Melzer et al. 2010; Wen et al. 2013), in adults and children living downstream of a PFOA manufacturing plant (Knox et al. 2011; Lopez-Espinosa et al. 2012; Steenland et al. 2011; Winquist and Steenland 2014), and in Korean adults (Ji et al. 2012). Sex-specific associations with THs have also been noted for other toxicants, including perchlorate (Blount et al. 2006) and BPA (Romano et al. 2015). The reasons for these differences by sex are not fully known, and may include random error; for PFASs, we speculate that sex-specific associations may relate to different toxicokinetics of PFASs in men and women (Harada et al. 2005), the effects of sex steroids on T4-binding globulin clearance in the liver (Tahboub and Arafah 2009) (which would affect T4 binding capacity in serum), and different distributions of TH stressors other than high TPOAb and low iodine status between the sexes (e.g., pregnancy).

### Strengths and Limitations

This study has several important strengths. We found associations between PFASs and THs in the subgroup of U.S. adults with high TPOAb and low urinary iodine. The high TPOAb and low iodine population represents a small but substantial fraction of U.S. adults (approximately 1.3%, or 3 million adults). However, the results are based on a small sample size (*n* = 26) and should be interpreted with caution. To the best of our knowledge, this is the first study to consider both iodine status and TPOAb status in an analysis of associations with THs of any environmental chemical. We controlled for many important covariates, including serum cotinine, a biomarker of tobacco exposure which has not always been considered in prior studies. But, we were unable to control for co-exposures to many other thyroid disrupting chemicals (e.g., serum PCBs, polybrominated diphenyl ethers, organochlorines, dioxins, or furans; urinary BPA or phthalates) because these chemicals were not measured in the same subset of 2007–2008 NHANES participants. In theory, cumulative exposures to other thyroid-disrupting chemicals may alter and possibly amplify the effects of PFASs or other thyroid toxicants on THs, especially in those with a diminished capacity to compensate. It would be worthwhile to test the “multiple hit” hypothesis with other chemicals, both individually and modeled as mixtures, in other vulnerable subpopulations.

As with any cross-sectional study, we cannot rule out the possibility that reverse causation may explain our results. Future studies should investigate the role of serum-binding protein levels, which may affect both PFAS and T4 levels, and urinary or blood selenium, an essential nutrient required for the metabolism of T4 to T3 by de-iodinase enzymes. Finally, recent evidence suggests that the fT4 radioimmunoassy used in this study may be somewhat sensitive to serum-binding protein levels, although any measurement error is expected to be less than with the analog methods used in many previous studies (Chang et al. 2007). Potential nonlinearities, and the potential for sex-specific differences in PFAS–TH associations also deserve further attention.

## Conclusions

We found evidence of modest PFAS-associated thyroid disruption in a subgroup of U.S. adults who had markers of both high TPOAb and low iodine. We estimate that approximately 1.3% of the U.S. adult population, or 3 million adults, fall into this potentially vulnerable subgroup. Small sample sizes limited the precision of the estimates in this segment of the population. Our findings are consistent with the “multiple hit hypothesis,” suggesting that individuals with multiple stressors on their thyroid systems may, on average, be more vulnerable to PFAS-induced thyroid disruption. These findings may have implications for other thyroid-disrupting chemicals, both individually and as aggregate exposures in complex mixtures. Future research on PFASs and other thyroid-disrupting chemicals should consider effect modification by thyroid system stressors, including TPOAb, iodine deficiency, and pregnancy, among others.

## Supplemental Material

(596 KB) PDF
