# Supplementary material for: Cross-Sectional Associations of Serum Perfluoroalkyl Acids and Thyroid Hormones in U.S. Adults: Variation According to TPOAb and Iodine Status (NHANES 2007–2008)
Source: Environ Health Perspect. 2015 Oct 30;124(7):935–42. doi: 10.1289/ehp.1409589 (PMC4937851; doi:10.1289/ehp.1409589)
Supplement: (596 KB) PDF [file ehp.1409589.s001.acco.pdf]

Environ Health Perspect
DOI: 10.1289/ehp.1409589

Note to readers with disabilities: *EHP* strives to ensure that all journal content is accessible to all readers. However, some figures and Supplemental Material published in *EHP* articles may not conform to 508 standards due to the complexity of the information being presented. If you need assistance accessing journal content, please contact ehp508@niehs.nih.gov. Our staff will work with you to assess and meet your accessibility needs within 3 working days.

Supplemental Material

Cross-Sectional Associations of Serum Perfluoroalkyl Acids and Thyroid Hormones in U.S. Adults: Variation According to TPOAb and Iodine Status (NHANES 2007-2008)

Glenys M. Webster, Stephen A. Rauch, Nathalie Ste Marie, Andre Mattman, Bruce P. Lanphear, and Scott A. Venners

Table of Contents

Figure S1. Directed Acyclic Graph (DAG) showing the causal relationships assumed among variables. All variables except BMI were included in the final models.

Table S1. Spearman correlations (rho) among PFASs and thyroid hormones in our study sample (n=1525 US adults, NHANES 2007-2008)

Figure S2. Percent differences in serum thyroid hormone levels for an interquartile ratio increase in serum PFAS concentrations in US adults (NHANES 2007-2008). Results are identical to those shown in Figure 3, but are re-grouped to allow for comparisons in the associations across thyroid stressors for each chemical. Results are stratified by TPOAb status (Normal = <9, High = ≥9 IU/mL serum) and iodine status (Normal = ≥100, Low = <100 µg/L urine). Results are shown for 4 groups: T0I0: Normal TPOAb, normal iodine (n=1012); T0I1: Low Iodine only (n=400); T1I0: High TPOAb only (n=87); T1I1: High TPOAb and Low Iodine (n=26). Error bars represent the 95% confidence intervals. Models are adjusted for age, race, log serum cotinine, sex, parity, pregnancy and menopause status. Interquartile ratios: 3.2 (PFHxS), 2.1 (PFNA), 2.1 (PFOA), 2.5

(PFOS). PFASs and THs were Ln-transformed in models. % differences = [(IQ Ratio^Beta)-1]*100

Table S2. Sex-specific % differences (and 95% Confidence Intervals) in serum thyroid hormones for an interquartile range increase in serum PFAS levels in US adults. Results are shown for 4 subgroups stratified by Iodine and Thyroid Peroxidase Antibody (TPOAb) status. Significant (p<0.05) associations are shown in bold text. Significantly different associations in men and women (p interaction <0.1 for PFAS*sex) are marked with *

Figure S3. Sex-specific % differences in serum thyroid hormone levels for an interquartile ratio increase in Ln serum PFAS concentrations in US adults (NHANES 2007-2008). Results are stratified by Thyroid Peroxidase Antibody (TPOAb) status (Normal: <9, High: ≥9 IU/mL serum) and iodine status (Normal ≥100, Low: <100 μg/L urine). Results are shown for 4 groups: T0I0: Normal TPOAb, normal iodine (n=586 men / 426 women); T0I1: Low Iodine only (n=188 men / 212 women); T1I0: High TPOAb only (n=32 men / 55 women); T1I1: High TPOAb and Low Iodine (n=7 men / 19 women). Error bars represent the 95% confidence intervals. Models are adjusteda for age, race, log serum cotinine, sex, parity, pregnancy and menopause status. Interquartile ratios: 3.2 (PFHxS), 2.1 (PFNA), 2.1 (PFOA), 2.5 (PFOS). PFASs and THs were Ln-transformed in models. % differences = [(IQ Ratio^Beta)-1]*100. *Significantly different associations in men and women (p interaction <0.1 for PFAS*sex)

Table S3. Comparison of T1I1 results with all participants (n=26) and with one influential T1I1 participant excluded (n=25). Percent differences and 95% Confidence Intervals (95% CI) in serum thyroid hormone levels for each interquartile ratio (IQ Ratio) increase in serum PFAS concentrations in US adults with both high TPOAb and low iodine

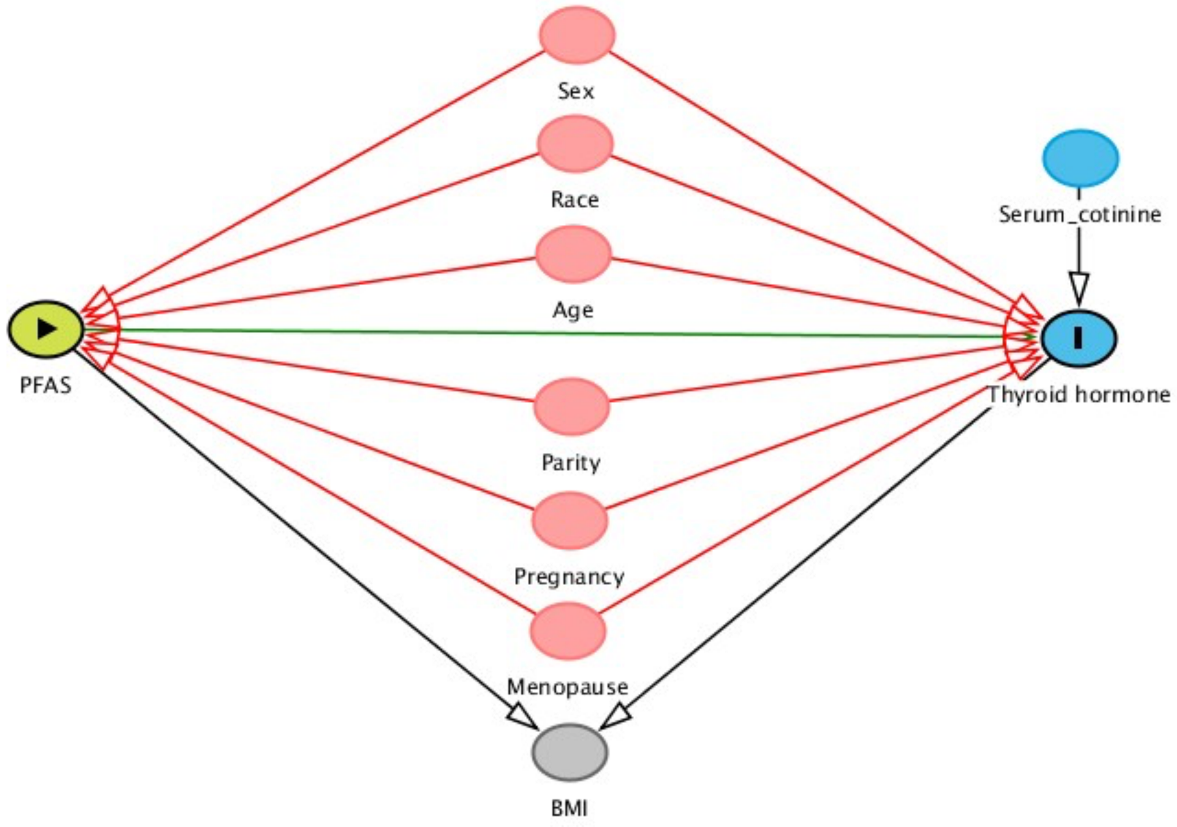

Figure S1. Directed Acyclic Graph (DAG) showing the causal relationships assumed among variables. All variables except BMI were included in the final models.

Table S1. Spearman correlations (rho) among PFASs and thyroid hormones in our study sample (n=1525 US adults, NHANES 2007-2008)

	PFHxS	PFNA	PFOA	PFOS	Free T3	Total T3	Free T4	Total T4	TSH	TPOAb	Iodine
PFHxS	1	0.41**	0.57**	0.67**	0.03	-0.01	0.02	-0.01	0.01	-0.07**	0.02
PFNA		1	0.63**	0.62**	0.04	-0.03	-0.02	-0.03	-0.06**	0.04	0.06**
PFOA			1	0.62**	0.07**	0.02	0.01	-0.05*	0.00	-0.05**	0.05**
PFOS				1	-0.09**	-0.12**	0.04*	-0.04	0.03	-0.02	0.03
Free T3					1	0.60**	0.11**	0.16**	-0.12**	0.07**	-0.03
Total T3						1	-0.01	0.3**	-0.04	0.08**	-0.02
Free T4							1	0.54**	-0.11**	-0.04*	-0.04*
Total T4								1	-0.08**	0.00	-0.06**
TSH									1	0.03	0.00
TPOAb										1	0.02
Iodine											1

* p≤0.1, ** p<0.05, using a 2-tailed test

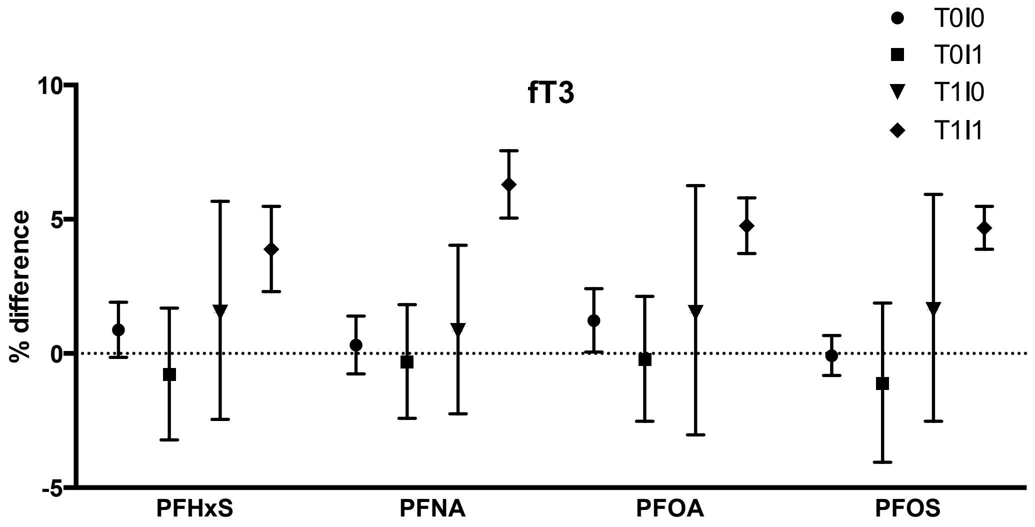
fT3
% difference
10
5
0
-5
PFHxS
PFNA
PFOA
PFOS
T0I0
T0I1
T1I0
T1I1

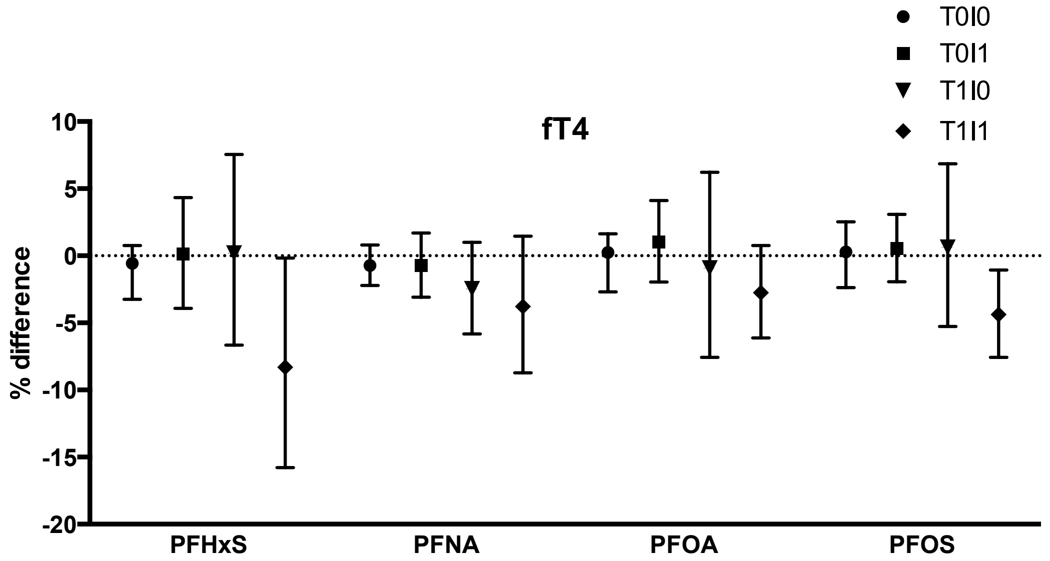
fT4
% difference
10
5
0
-5
-10
-15
-20
PFHxS
PFNA
PFOA
PFOS
T0I0
T0I1
T1I0
T1I1

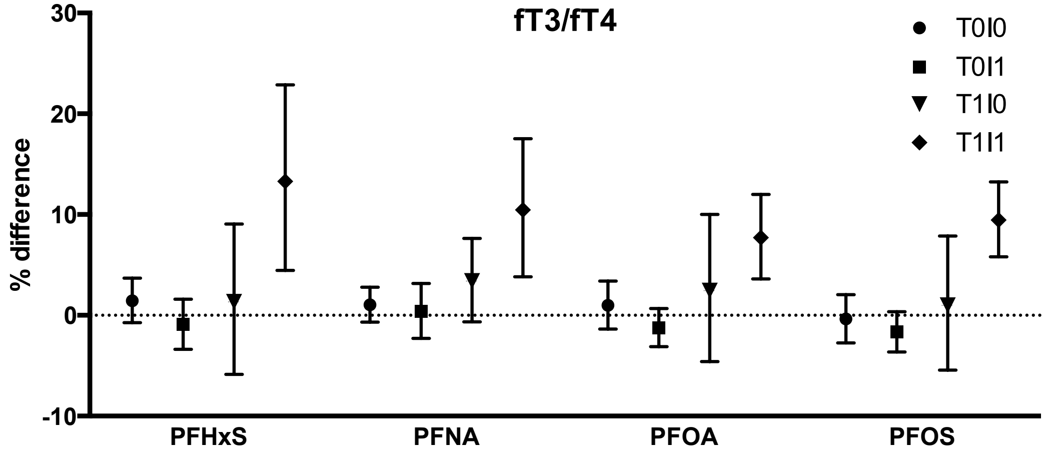
fT3/fT4
% difference
30
20
10
0
-10
PFHxS
PFNA
PFOA
PFOS
T0I0
T0I1
T1I0
T1I1

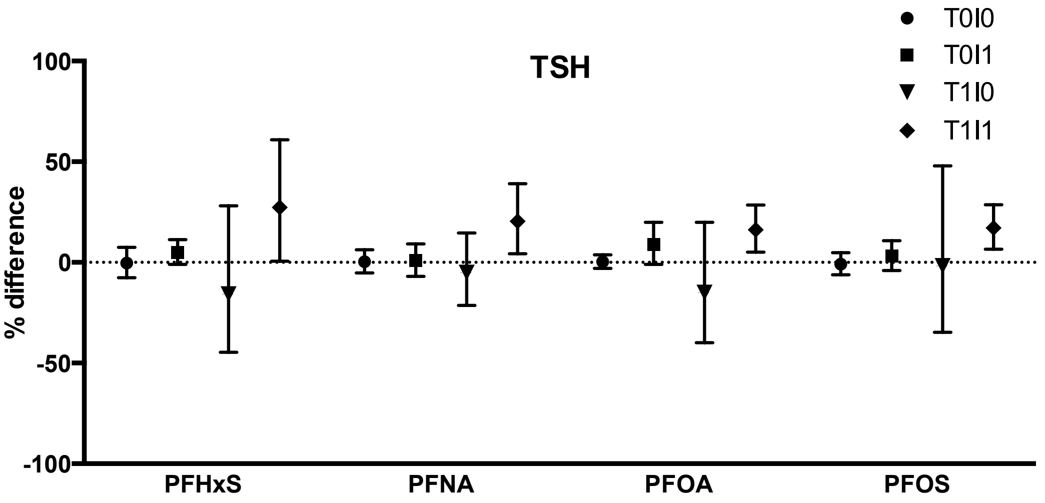
TSH
T0I0
T0I1
T1I0
T1I1
100
50
0
-50
-100
% difference
PFHxS
PFNA
PFOA
PFOS

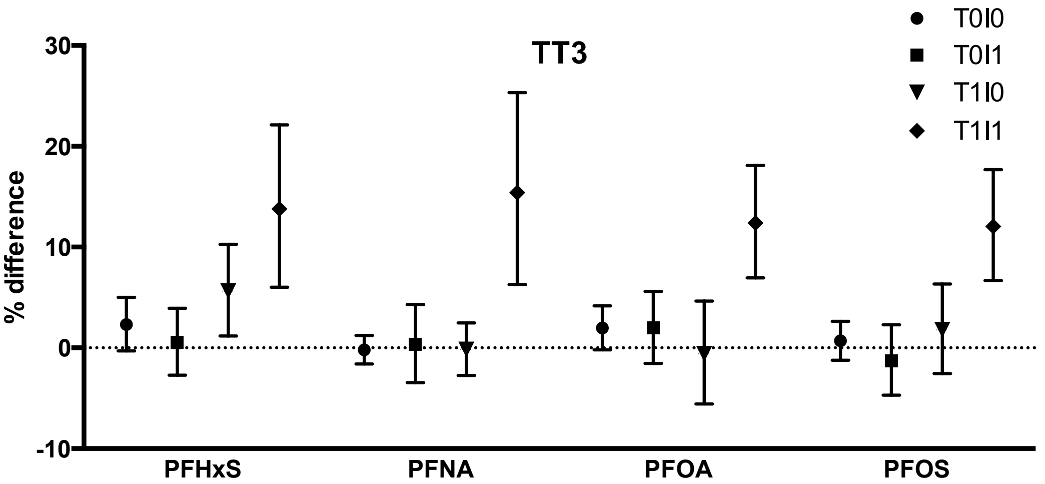
TT3
T0I0
T0I1
T1I0
T1I1
30
20
10
0
-10
% difference
PFHxS
PFNA
PFOA
PFOS

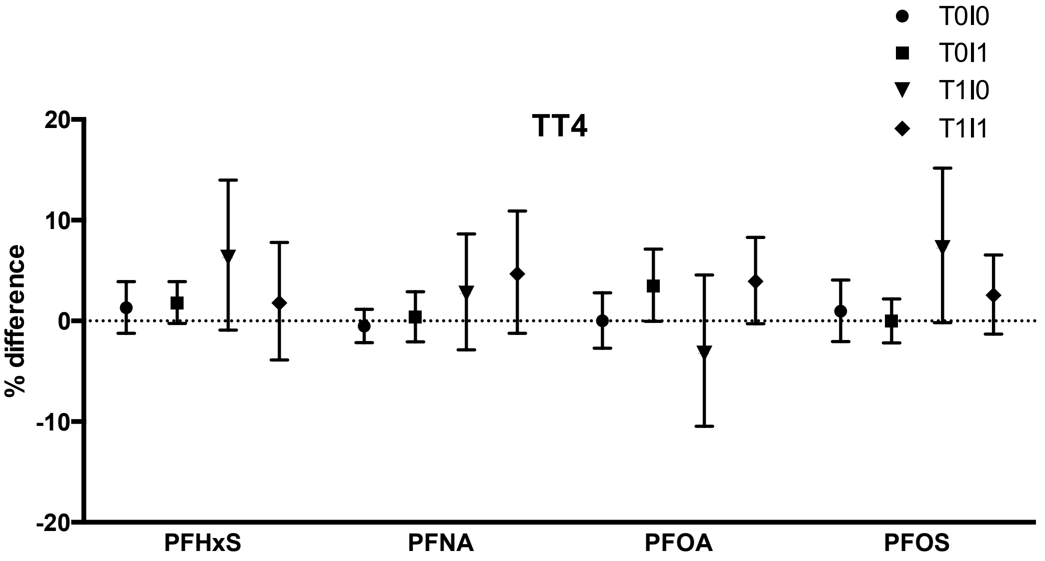
TT4
T0I0
T0I1
T1I0
T1I1
20
10
0
-10
-20
% difference
PFHxS
PFNA
PFOA
PFOS

Figure S2. Percent differences in serum thyroid hormone levels for an interquartile ratio increase in serum PFAS concentrations in US adults (NHANES 2007-2008). Results are identical to those shown in Figure 3, but are re-grouped to allow for comparisons in the associations across thyroid stressors for each chemical. Results are stratified by TPOAb status (Normal = <9, High = ≥9 IU/mL serum) and iodine status (Normal = ≥100, Low = <100 μg/L urine). Results are shown for 4 groups: T0I0: Normal TPOAb, normal iodine (n=1012); T0I1: Low Iodine only (n=400); T1I0: High TPOAb only (n=87); T1I1: High TPOAb and Low Iodine (n=26). Error bars represent the 95% confidence intervals. Models are adjusted for age, race, log serum cotinine, sex, parity, pregnancy and menopause status. Interquartile ratios: 3.2 (PFHxS), 2.1 (PFNA), 2.1 (PFOA), 2.5 (PFOS). PFASs and THs were Ln-transformed in models. % differences = [(IQ Ratio^Beta)-1]*100

Table S2. Sex-specific % differences[a] (and 95% Confidence Intervals) in serum thyroid hormones for an interquartile range increase in serum PFAS levels in US adults[a,b]. Results are shown for 4 subgroups stratified by Iodine and Thyroid Peroxidase Antibody (TPOAb) status[c]. Significant ($p<0.05$) associations are shown in bold text. Significantly different associations in men and women (p interaction <0.1 for PFAS*sex) are marked with *

		**T0I0[e]**		**T0I1[e]**		**T1I0[e]**		**T1I1[e]**	
**PFAS**	**TH**	**Men** (n=586)	**Women** (n=426)	**Men** (n=188)	**Women** (n=212)	**Men** (n=32)	**Women** (n=55)	**Men** (n=7)	**Women** (n=19)
PFHxS	fT3	0.4 (-0.5, 1.3)	1.6 (-0.7, 4.1)	0.1 (-1.8, 2.0)	-1.4 (-5.4, 2.7)	3.0 (-6.4, 13.3)	0.9 (-3.5, 5.5)	**-7.6 (-8.7, -6.5)**	**7.8 (5.8, 9.8)***
PFNA	fT3	-0.3 (-1.7, 1.1)	1.0 (-0.2, 2.4)	-0.9 (-3.6, 1.9)	0.2 (-2.0, 2.4)	0.5 (-7.6, 9.4)	0.9 (-2.0, 4.0)	-9.7 (-21.0, 3.2)	**6.8 (4.9, 8.8)***
PFOA	fT3	0.6 (-0.7, 2)	**2.1 (0.5, 3.8)**	-0.2 (-3.2, 3.0)	-0.2 (-3.2, 2.8)	2.8 (-3.8, 9.8)	0.9 (-3.8, 5.9)	**-6.7 (-12.8, -0.2)**	**5.1 (4.2, 6.1)***
PFOS	fT3	-0.1 (-1.1, 0.9)	0.0 (-1.8, 1.9)	-1.2 (-3.9, 1.7)	-1.1 (-4.6, 2.5)	1.0 (-5.0, 7.3)	1.9 (-2.3, 6.2)	**-7.1 (-12.9, -0.9)**	**5.1 (4.1, 6.1)***
PFHxS	fT4	**-1.8 (-3.6, 0.0)**	1.4 (-1.9, 4.7)*	1.1 (-2.8, 5.2)	-0.6 (-5.0, 4.1)	-0.8 (-18.1, 20.0)	0.6 (-5.4, 7.1)	**-26.9 (-33.0, -20.4)**	-1.5 (-7.1, 4.5)*
PFNA	fT4	-1.0 (-2.7, 0.8)	-0.4 (-2.4, 1.6)	-0.2 (-3.4, 3.0)	-1.1 (-4.1, 1.9)	-8.1 (-22.7, 9.1)	-1.0 (-5.1, 3.2)	**-40.7 (-63.9, -2.6)**	-2.4 (-5.3, 0.7)*
PFOA	fT4	-0.6 (-2.8, 1.5)	1.5 (-1.9, 5.0)	4.1 (-1.1, 9.7)	-1.2 (-4.4, 2.0)*	-1.9 (-10.1, 7.1)	-0.5 (-8.9, 8.7)	-20.0 (-40.1, 6.7)	-2.2 (-5.1, 0.9)
PFOS	fT4	-0.5 (-3.2, 2.4)	1.5 (-1.1, 4.1)	0.4 (-1.8, 2.7)	0.7 (-2.9, 4.3)	-1.4 (-12.0, 10.6)	1.4 (-5.3, 8.6)	**-31.5 (-45.7, -13.6)**	**-3.2 (-5.5, -1)***
PFHxS	fT3/fT4	**2.2 (0.2, 4.3)**	0.3 (-4.0, 4.7)	-1.0 (-4.4, 2.4)	-0.8 (-3.6, 2.0)	3.8 (-16.0, 28.3)	0.3 (-5.8, 6.7)	**26.5 (16.6, 37.2)**	**9.4 (1.8, 17.6)***
PFNA	fT3/fT4	0.7 (-1.3, 2.7)	1.5 (-0.7, 3.7)	-0.7 (-4.5, 3.3)	1.3 (-1.4, 4.1)	9.4 (-5.0, 26.0)	2.0 (-1.9, 6.0)	**52.3 (1.4, 128.7)**	**9.4 (4.4, 14.6)***
PFOA	fT3/fT4	1.3 (-1.6, 4.2)	0.6 (-2.6, 3.9)	**-4.1 (-6.7, -1.6)**	1.0 (-1.5, 3.6)*	4.8 (-5.7, 16.4)	1.4 (-6.6, 10.2)	16.7 (-8.7, 49.1)	**7.5 (3.7, 11.3)**
PFOS	fT3/fT4	0.3 (-2.6, 3.3)	-1.4 (-4.1, 1.4)	-1.6 (-4.0, 0.9)	-1.8 (-4.5, 1.0)	2.4 (-9.3, 15.5)	0.5 (-6.5, 7.9)	**35.6 (12.4, 63.6)**	**8.6 (5.8, 11.5)***
PFHxS	TSH	1.1 (-8.3, 11.5)	-2.4 (-10, 5.9)	9.2 (-2.5, 22.2)	2.1 (-7.3, 12.5)	-15.1 (-44.8, 30.7)	-16.2 (-51.9, 46.1)	**65.7 (25.6, 118.8)**	17.1 (-4.6, 43.8)*
PFNA	TSH	-0.8 (-11, 10.7)	1.7 (-6.9, 11.1)	5.6 (-10.8, 24.9)	-2.9 (-14, 9.5)	7.6 (-31, 67.7)	-7.9 (-22.1, 9)	**275.1 (73.5, 711.3)**	**16.4 (4.0, 30.4)***
PFOA	TSH	-0.3 (-6.7, 6.6)	1.2 (-7.1, 10.3)	11.9 (-6.4, 33.8)	6.8 (-3.1, 17.6)	0.1 (-23.8, 31.4)	-21.0 (-51.1, 27.6)	**141.4 (51.6, 284.3)**	**13.7 (5.7, 22.2)***
PFOS	TSH	-1.6 (-10.6, 8.4)	0.4 (-8.2, 9.7)	7.4 (-6.0, 22.8)	-1.2 (-9.4, 7.9)	11.7 (-22.9, 61.9)	-6.5 (-42.4, 51.6)	**188.4 (131.4, 259.4)**	**13.5 (8.7, 18.4)***
PFHxS	TT3	1.6 (-1.0, 4.4)	3.3 (-0.2, 7.0.0)	**-4.8 (-8.2, -1.2)**	**4.5 (0.9, 8.2)***	3.0 (-6.9, 13.8)	**6.8 (2.1, 11.8)**	0.9 (-11.7, 15.3)	**18.2 (7.1, 30.5)***
PFNA	TT3	-1.8 (-3.7, 0.2)	1.8 (-0.8, 4.4)*	-2.9 (-6.3, 0.6)	3.1 (-1.7, 8.2)*	-6.0 (-14.2, 2.9)	1.3 (-2.7, 5.5)	-9.8 (-27.4, 12)	**16.3 (5.7, 27.9)***
PFOA	TT3	1.0 (-1.2, 3.2)	**3.4 (0.2, 6.6)**	-2.8 (-6.0, 0.4)	**5.7 (0.8, 10.9)***	-4.0 (-15.0, 8.3)	1.0 (-5, 7.3)	**18.6 (8.1, 30.0)**	**12.2 (6.7, 18.0)**
PFOS	TT3	0.4 (-1.6, 2.5)	1.1 (-1.9, 4.2)	**-4.0 (-7.2, -0.6)**	1.6 (-2.8, 6.3)*	-7.0 (-13.6, 0.1)	5.4 (-0.8, 12.1)*	12.1 (-1.0, 26.9)	**12.0 (6.4, 17.9)**
PFHxS	TT4	-0.2 (-3.2, 3.0)	3.5 (-0.5, 7.7)	-0.8 (-3.6, 2.1)	**3.6 (0.3, 7.1)***	9.3 (-1.3, 21)	5.0 (-3.1, 13.7)	**-18.0 (-28.6, -5.9)**	**9.0 (2.3, 16.1)***
PFNA	TT4	-2.2 (-5.0, 0.6)	1.7 (-0.4, 3.8)*	0.2 (-3.3, 3.9)	0.5 (-4.3, 5.5)	-0.2 (-9.7, 10.3)	3.4 (-2.6, 9.8)	**-39.4 (-52.8, -22.3)**	6.4 (-1.2, 14.5)*
PFOA	TT4	-0.5 (-3.6, 2.7)	0.7 (-3.8, 5.5)	2.9 (-2.2, 8.3)	3.9 (-0.6, 8.6)	-3.4 (-9.4, 3.1)	-3.2 (-13.0, 7.8)	-2.9 (-19.5, 17.2)	4.1 (-0.3, 8.8)
PFOS	TT4	0.9 (-3.1, 5.1)	1.0 (-1.9, 4.1)	-1.4 (-3.3, 0.6)	1.4 (-2.2, 5.2)	0.2 (-7.5, 8.6)	**10.1 (0.9, 20.1)***	**-17.5 (-31.2, -1.1)**	3.3 (-1.4, 8.3)*

a. PFASs and THs were Ln-transformed in models. % differences = [(IQ Ratio^Beta)-1]*100
b. Interquartile ratio = 75th / 25th percentiles of serum PFASs: 3.2 (PFHxS), 2.1 (PFNA), 2.1 (PFOA), 2.5 (PFOS)
c. Models are adjusted for age, race, log serum cotinine, sex, parity, pregnancy and menopause status, and included a PFAS*sex interaction term
d. TPOAb cutoffs: Normal: <9, High: ≥9 IU/mL serum. Iodine cutoffs: Normal ≥100, Low: <100 µg/L urine
e. T0I0: Normal TPOAb and iodine; T0I1: Low iodine only; T1I0: High TPOAb only; T1I1: High TPOAb and low iodine

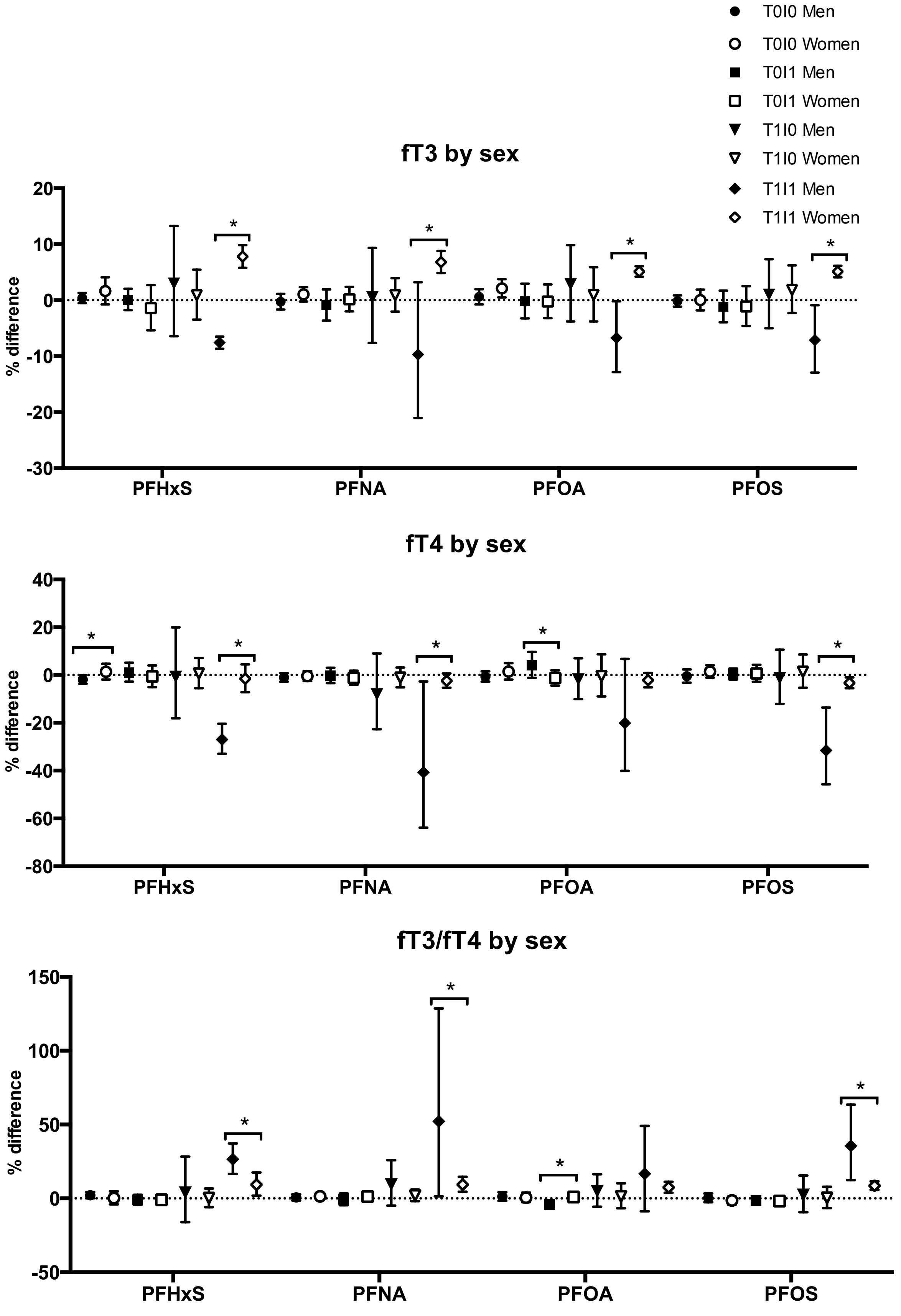
T0I0 Men
T0I0 Women
T0I1 Men
T0I1 Women
T1I0 Men
T1I0 Women
T1I1 Men
T1I1 Women
fT3 by sex
% difference
20
10
0
-10
-20
-30
PFHxS
PFNA
PFOA
PFOS
fT4 by sex
% difference
40
20
0
-20
-40
-60
-80
PFHxS
PFNA
PFOA
PFOS
fT3/fT4 by sex
% difference
150
100
50
0
-50
PFHxS
PFNA
PFOA
PFOS

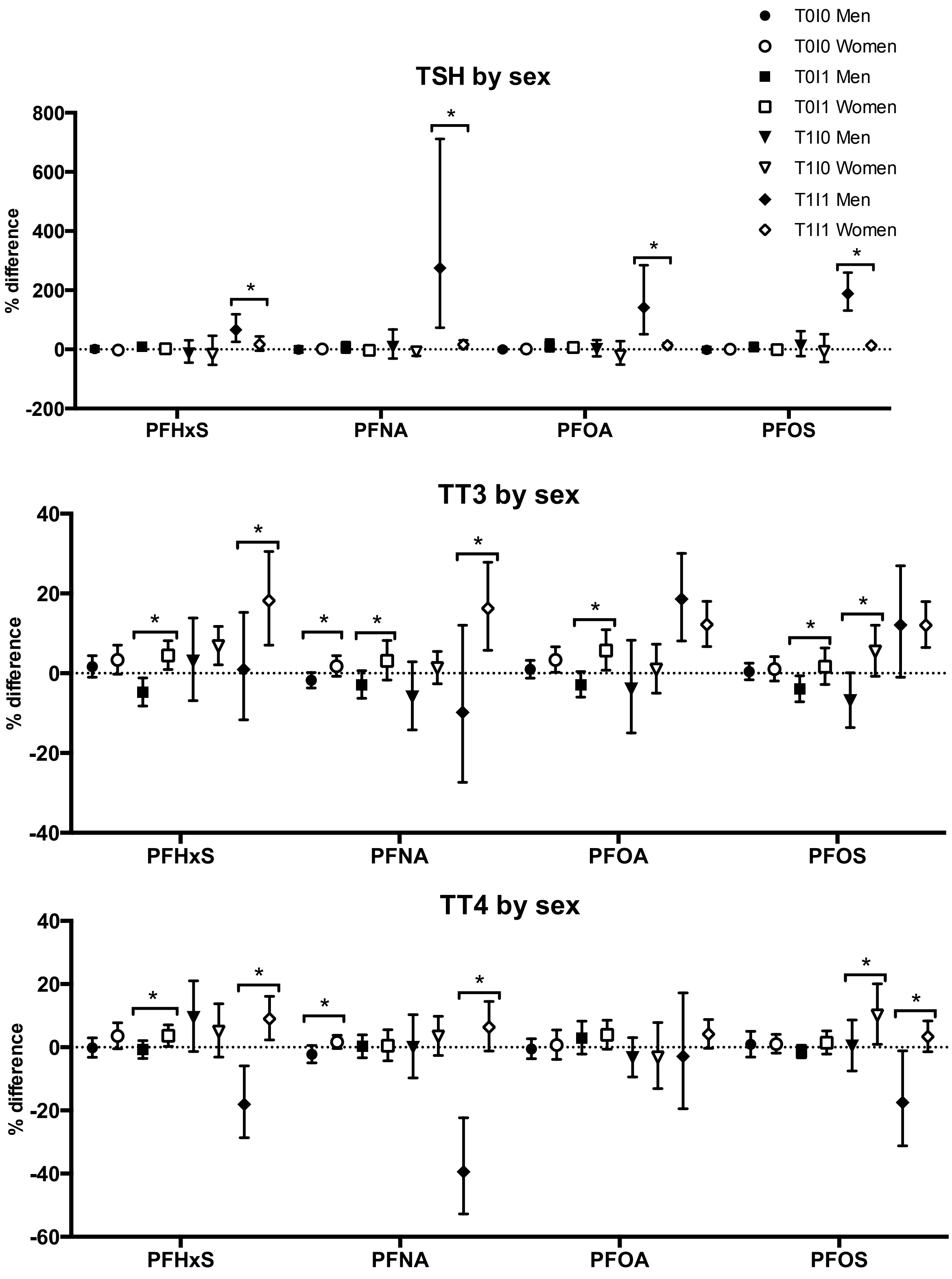
T0I0 Men
T0I0 Women
T0I1 Men
T0I1 Women
T1I0 Men
T1I0 Women
T1I1 Men
T1I1 Women
TSH by sex
% difference
800
600
400
200
0
-200
PFHxS
PFNA
PFOA
PFOS
TT3 by sex
% difference
40
20
0
-20
-40
PFHxS
PFNA
PFOA
PFOS
TT4 by sex
% difference
40
20
0
-20
-40
-60
PFHxS
PFNA
PFOA
PFOS

Figure S3. Sex-specific % differences in serum thyroid hormone levels for an interquartile ratio increase in Ln serum PFAS concentrations in US adults (NHANES 2007-2008). Results are stratified by Thyroid Peroxidase Antibody (TPOAb) status (Normal: <9, High: ≥9 IU/mL serum) and iodine status (Normal ≥100, Low: <100 µg/L urine). Results are shown for 4 groups: T0I0: Normal TPOAb, normal iodine (n=586 men / 426 women); T0I1: Low Iodine only (n=188 men / 212 women); T1I0: High TPOAb only (n=32 men / 55 women); T1I1: High TPOAb and Low Iodine (n=7 men / 19 women). Error bars represent the 95% confidence intervals. Models are adjusteda for age, race, log serum cotinine, sex, parity, pregnancy and menopause status. Interquartile ratios: 3.2 (PFHxS), 2.1 (PFNA), 2.1 (PFOA), 2.5 (PFOS). PFASs and THs were Ln-transformed in models. % differences = [(IQ Ratio^Beta)-1]*100. *Significantly different associations in men and women (p interaction <0.1 for PFAS*sex)

Table S3. Comparison of T1I1 results with all participants (n=26) and with one influential T1I1 participant excluded (n=25). Percent differences[a] and 95% Confidence Intervals (95% CI) in serum thyroid hormone levels for each interquartile ratio (IQ Ratio) increase in serum PFAS concentrations[b,c] in US adults with both high TPOAb and low iodine[d]

		All T1I1 participants (n=26)		1 influential T1I1 participant excluded[f] (n=25)	
TH	PFAS	% difference[a] (95% CI)		% difference[a] (95% CI)	
fT3	PFHxS	3.9 (2.3, 5.5)	**	-3.5 (-6.7, -0.1)	**
	PFNA	6.3 (5.0, 7.5)	**	-3.3 (-8.1, 1.8)	
	PFOA	4.8 (3.7, 5.8)	**	1.8 (-3.4, 7.3)	
	PFOS	4.7 (3.9, 5.5)	**	0.0 (-8.5, 9.2)	
fT4	PFHxS	-8.3 (-15.8, -0.2)	**	-15.8 (-29, -0.2)	**
	PFNA	-3.8 (-8.7, 1.4)		-7.9 (-28.2, 18.2)	
	PFOA	-2.7 (-6.1, 0.8)		-8.3 (-24.8, 11.8)	
	PFOS	-4.4 (-7.6, -1.1)	**	-24.1 (-36.8, -8.8)	**
fT3/fT4	PFHxS	13.3 (4.4, 22.9)	**	14.7 (-2.8, 35.5)	*
	PFNA	10.5 (3.8, 17.5)	**	5.0 (-19.4, 36.8)	
	PFOA	7.7 (3.6, 12.0)	**	11.0 (-7.8, 33.7)	
	PFOS	9.5 (5.8, 13.2)	**	31.7 (15.7, 49.8)	**
TSH	PFHxS	27.3 (0.7, 60.9)	**	39.6 (-8.8, 113.8)	
	PFNA	20.5 (4.3, 39.1)	**	30.0 (-16.3, 101.7)	
	PFOA	16.2 (5.1, 28.5)	**	52.5 (1.4, 129.3)	**
	PFOS	17.1 (6.6, 28.7)	**	82.3 (12.5, 195.3)	**
TT3	PFHxS	13.8 (6.0, 22.1)	**	4.0 (-9.8, 19.8)	
	PFNA	15.4 (6.3, 25.3)	**	-1.9 (-18.6, 18.2)	
	PFOA	12.4 (7.0, 18.1)	**	16.0 (7.4, 25.4)	**
	PFOS	12.0 (6.7, 17.7)	**	13.9 (4.0, 24.7)	**
TT4	PFHxS	1.8 (-3.9, 7.8)		-6.8 (-16.8, 4.5)	
	PFNA	4.7 (-1.2, 10.9)		-7.1 (-17.9, 5.0)	
	PFOA	3.9 (-0.3, 8.3)		0.0 (-12.2, 13.9)	
	PFOS	2.5 (-1.3, 6.5)		-13.7 (-21.1, -5.5)	**

a. PFASs and THs were Ln-transformed in models. % differences = [(IQ Ratio^Beta)-1]*100
b. Interquartile ratio (IQ Ratio) = 75th / 25th percentiles of serum PFASs: 3.2 (PFHxS), 2.1 (PFNA), 2.1 (PFOA), 2.5 (PFOS)
c. Models are adjusted for age, race, log serum cotinine, sex, parity, pregnancy and menopause status
d. TPOAb cutoffs: Normal: <9, High: ≥9 IU/mL serum. Iodine cutoffs: Normal ≥100, Low: <100 µg/L urine
e. T1I1: High TPOAb and low iodine
f. Participants with DF Beta >2.0 were considered influential; one participant met this criterion in 9 T1I1 models. This participant was a 55 year old, white, nulliparous and post-menopausal woman, with low serum PFAS and cotinine levels (all <LOD) and low fT3 and TT3 levels (<5th percentile). Her fT4, TT4 and TSH levels were all in the 25th-75th percentile range. Her TPOAb and iodine levels were in the >95th percentile and <5th percentile, respectively

*p<0.1; ** p<0.05
